# Protein-Based Films and Coatings for Food Industry Applications

**DOI:** 10.3390/polym13050769

**Published:** 2021-03-02

**Authors:** Vlad Mihalca, Andreea Diana Kerezsi, Achim Weber, Carmen Gruber-Traub, Jürgen Schmucker, Dan Cristian Vodnar, Francisc Vasile Dulf, Sonia Ancuța Socaci, Anca Fărcaș, Carmen Ioana Mureșan, Ramona Suharoschi, Oana Lelia Pop

**Affiliations:** 1 Department of Food Science, University of Agricultural Science and Veterinary Medicine, 400372 Cluj-Napoca, Romania; vlad.mihalca@yahoo.com (V.M.); andreeadianakerezsi@gmail.com (A.D.K.); dan.vodnar@usamvcluj.ro (D.C.V.); sonia.socaci@usamvcluj.ro (S.A.S.); anca.farcas@usamvcluj.ro (A.F.); carmen.muresan@usamvcluj.ro (C.I.M.); 2 TERRA Research and Teaching Center, Gembloux Agro-Bio Tech, University of Liege, Department of Food Science and Formulation, Passage des Deportes-2, B-5030 Gembloux, Belgium; 3 Fraunhofer Institute for Interfacial Engineering and Biotechnology IGB, Nobelstraße 12, 70569 Stuttgart, Germany; achim.weber@igb.fraunhofer.de (A.W.); carmen.gruber-traub@igb.fraunhofer.de (C.G.-T.); juergen.schmucker@igb.fraunhofer.de (J.S.); 4 Department of Biochemistry, University of Agricultural Sciences and Veterinary Medicine, 400372 Cluj-Napoca, Romania; francisc.dulf@usamvcluj.ro

**Keywords:** food package, protein, protein films, protein coatings, functionalization, antioxidant, antimicrobial, antifungal

## Abstract

Food packaging is an area of interest not just for food producers or food marketing, but also for consumers who are more and more aware about the fact that food packaging has a great impact on food product quality and on the environment. The most used materials for the packaging of food are plastic, glass, metal, and paper. Still, over time edible films have become widely used for a variety of different products and different food categories such as meat products, vegetables, or dairy products. For example, proteins are excellent materials used for obtaining edible or non-edible coatings and films. The scope of this review is to overview the literature on protein utilization in food packages and edible packages, their functionalization, antioxidant, antimicrobial and antifungal activities, and economic perspectives. Different vegetable (corn, soy, mung bean, pea, grass pea, wild and Pasankalla quinoa, bitter vetch) and animal (whey, casein, keratin, collagen, gelatin, surimi, egg white) protein sources are discussed. Mechanical properties, thickness, moisture content, water vapor permeability, sensorial properties, and suitability for the environment also have a significant impact on protein-based packages utilization.

## 1. Introduction

Since ancient times, humans have tried to find optimal solutions for packing their food products as easily and productively as possible. Along with technological advancements, society found better and more resistant materials to improve food packing. As time passed by, the population started to develop alternatives when discussing packaging. In this regard, the research for new, unconventional materials that can be used for packaging food in a more sustainable and eco-friendly way has seen a constant increase in the last few decades. Packaging has many duties such as: to preserve substances against contamination and perishability, to move easily and keep goods, and to give a constant measure of the contents. A package has three important tasks: to protect the contents, to give good marketing to a product, and to deliver useful information to the customer. A fourth purpose is related to advertisement because easy to use packaging increases market opportunities. Thus, four significant functions of packaging have been identified: security, containment, communication and accessibility. All these attributes are interconnected, and all have to be evaluated and taken into consideration within the process of packaging development [1].

The objective of the present paper is to provide an overview of the recent progress regarding the applicability of animal and vegetal derived proteins in developing food (edible) films, coatings, and innovative packaging materials. Protein characteristics, suitability, protein-based functionality, and economic aspects are also discussed.

## 2. Materials Used for Protein-Based Packaging and Edible Packaging

Over the years, humans started developing different kinds of films and coatings meant to protect food against spoilage. For example, in the 12th century fruits were coated in a wax layer to slow down the water loss process and during, the 15th century, a Japanese scientist named Yuba discovered the first edible film from soymilk [2].

Starting in the 16th century in Europe, enough people figured out how to use different substances in order to collect products and to prevent alteration, by reducing the moisture and covering the meat with oil. In the 19th century, they started coating food in oil and gelatin. In the same period, foods like hazelnuts, nuts and almonds were coated with sucrose, to prevent rancidness and oxidation [3].

In order to have a waste disposal value close to zero, there is edible packaging made from ingredients that can be eaten alongside the contained food or beverage, the edible packaging and the food forming together a constant and cohesive system [4]. An edible package is defined as a thin layer that covers the food surface. In the beginning, it was called edible coating and afterward edible film. If it is not consumed, the edible film degrades very fast and in this way is reduces the demands of landfills, in comparison with plastic and biodegradable products [5].

The quality of the food might be improved by using edible films and coatings; the film’s or coating’s composition having a great role in this process. According to Pavli et al., 2018, flavorings, antioxidants, antimicrobials, probiotics and nanomaterials are known to be beneficial in enhancing the quality of food [6]. For the development of an effective edible package, the selective permeability and gas barrier are specific conditions [7].

These thin layers of edible substances are created between food parts or on the surface. They have different properties, from controlling oxygen, carbon dioxide, taste and aroma between other food parts or the surrounding atmosphere to the capability of carrying a various array of food additives as preservatives, antimicrobial agents and antioxidants. They could offer all these functional properties as a packaging material if they are prepared in the correct way [3].

Regarding the edible food packaging, polysaccharides are a great option with respect to edible packaging material. To reduce conventional plastic packaging, starch, xanthan gum, carrageenan, pectin and alginate (polysaccharides) were used to produce edible films and coatings, because they are natural products, with low toxicity and selective permeability for oxygen and carbon dioxide. All these attributes of the coatings and edible films made from polysaccharides can prolong fruit shelf life [8]. Brown algae (*Phaeophyceae*) contains a polysaccharide named alginate. This polysaccharide consists of α-L-guluronate (G) and R-D-mannuronate (M) bonds in the (1-4) chain [8,9]. Another polysaccharide with microbial properties is pullulan, composed of maltotriose and α (1,6) glycosidic units, produced by *Aureobasidium pullulans* from starch [10]. Pullulan is water-soluble, it has no color, smell and taste, and is also an oil permeable and heat sealable edible film [8].

A very important question to ask is how can we obtain almost unlimited amounts of prime ingredients? The answer in the case of polysaccharides is by looking back at what nature has to offer. Cellulose is the earth’s most abundant organic compound, and scientists rapidly found ways to procure plant origin polysaccharides, marine origin polysaccharides and even microbial polysaccharides [11]. An excellent cellulose compound with thermal gelatinization and water-soluble characteristics film-forming is the (carboxymethyl cellulose (CMC) [8].

Researchers started to study and develop nanostructured antimicrobial edible films in order to further protect and contain the food matrix [12].

Overtime, edible films have become widely used for a variety of different products and different food categories such as meat products, vegetables or dairy products.

According to Farhan et al., 2020, the edible film of semi-refined κ-carrageenan can be developed by a water extract from the process of fenugreek seed germination. For fresh chicken breast this edible film can be used as an alternative to conventional plastic films that are used in the packaging of chicken meat [13,14]. Furcellaran, a genus of red algae, is one of the most important sources of carrageenan. Jamróz et al. used furcellaean with nanofillers, nanoparticles of maghemite, and graphene oxide with good antimicrobial activity (for the nanofillers film), but not excellent mechanical properties [15].

Three types of coating mixture using carboxymethyl cellulose, sodium alginate and carrageenan were used for the effective protection of cherry tomatoes. These coverings of the tomatoes, in combination with preservation in a controlled atmosphere, were used to validate the preservation system. The carrageenan edible film was proven protect the cherry tomatoes, from where it can be used for other vegetables and fruits as preservation packaging [16].

According to Cruz-Diaz et al., 2019, protein-based films treated with ultrasound have lower water-vapor permeability than the films treated with heat. Only the color of the protein-based film was affected by addition of microbial transglutaminase into the solutions treated with ultrasound, while the properties of the film were not affected. Another study with whey protein films has favorable results for cheese slices and more studies will be continued on this subject [17].

Furthermore, protein-based packages may be an active package. The interaction of the package (or of one of the ingredients) with the packaged food or the nearby environment makes it active. The most common ingredients used in order to obtain an active film or coating are antioxidant and antibacterial compounds. Delaying the oxidation (by binding pro-oxidation compounds or by releasing antioxidant ones) and stopping pathogen development (organic acids, negatively charged phosphate groups, essential oils, anthocyanins, chitosan) are, in the stated cases, the main objectives of the active packages [18,19]. Chemical, biochemical or biological changes on the surface of the product turn on the release of the active compounds and ensure a prolonged freshness and shelf life.

**Figure 1:**
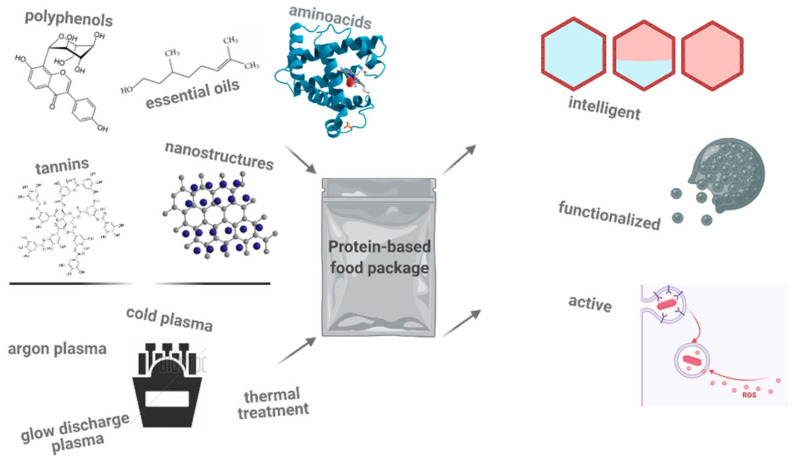
Compounds and technologies that add values to protein-based food packages.

Food packaging is a broad area where innovation has no limit. An important step was made in the food sector when intelligent and smart packages were applied. Intelligent packages equipped with sensors, indicators (pH, temperature), or tagged with radio frequency inform the consumer based on the ability of the package to feel, notice, or record outer or inner changes in the product. These systems are really advanced, using computer applications, nanotechnologies and microelectronics [20,21]. In Figure 1 multiple valences that a protein-based food package can have can be seen.

## 3. Proteins Used for Food Films or Coatings

Many researchers have dedicated their work to the insertion of biopolymers in active packaging. Proteins, units formed by a covalent peptide bond, are among these biopolymers [22]. Many important protein sources are found in different vegetable or animal sources. Because of the widespread of resources within these basic products, researchers started to extract polypeptides from a large variety of vegetable and animal products or by-products [23,24]. For example, *Moringa oleifera* seeds are an important source of proteins (40%) with antioxidant activity [25]; insects are also an explored source together with fish by-products [26] or plant-based by-products [27].

There are different types of proteins (Table 1) such as the plant-derived proteins from corn [28], wheat and soy [28], etc., and animal-derived proteins such as collagen [29], keratin [30], casein [31] and gelatin [32,33].

### 3.1. Animal Protein-Based Packages

When purchasing meat consumers associate color with freshness and thus color is an important quality attribute. Therefore, an important characteristic of the film applied to meat products is the transparency [41]. Bacterial alteration may give color changes in meat products. Thus, application of active packages, that contain antibacterial molecules (e.g., essential oils, antibacterial proteins, nanoparticles), may be useful [42,43,44]. For example, in refrigerated chicken breast meat the growth of bacteria responsible for spoilage has been controlled by a whey protein coating incorporated with oregano essential oil [7].

Milk proteins have the ability to form malleable, transparent and tasteless films. Milk proteins are classified as caseins and whey proteins. Caseins can form films which are stable at different pH, temperature and salt levels. These casein films can be carriers of food additives, i.e., antioxidants, food colors or antibacterial compounds. The edible casein films can be prepared from aqueous caseinate solutions which are finally dried [8]. Moreover, polymerized whey proteins could be applied as a thickening agent for increasing viscosity and decreasing syneresis of yogurt and can be directly obtained from Cheddar cheese whey by employing membrane separation [45].

Braber [46] and his colleagues obtained an antifungal whey-based film, with excellent properties against *Aspergillus niger* (10^3^ spores/mL), but not against *Penicillium roqueforti*. Chitosan was incorporated in low quantity and, as a plasticizer, glycerol was utilized. In order to neutralize the chitosan charges, sodium tripolyphosphate was added.

Interesting results showed that whey-based films can be formed with excellent water permeability and good flexibility if almond oil is incorporated into the film formation matrix [47].

In order to meet some of disadvantages of packages obtained from natural compounds (i.e., poor water resistance), researchers innovate by also using natural bioactive molecules. For example, a polyphenolic compound, tannic acid, was used as crosslinking for casein films [48].

Another animal protein, frequently used in (edible) food packages is gelatin, obtained from the hydrolysis of collagen. Fish, pork and bovine are different sources of collagen for gelatin production. Films based on bovine gelatin were shown to possess a hydrophobic surface, and the inclusion of chitin in the gelatin film increases the hydrophobicity even more [49]. A fish gelatin film, obtained by compression with added anthocyanins, extracted from red cabbage, showed not only antioxidant activity—as was expected—but also better mechanical and water resistance [50].

Collagen, the most abundant protein in the body, is versatile for multiple applications [51,52]. In packaging applications, collagen is usually used with other biopolymers. An example is the agar–alginate–collagen film with incorporated silver nanoparticles, with excellent antimicrobial properties (against *Listeria monocytogenes* and *Escherichia coli*) and good mechanical and water resistance [29].

In a study from 2017, collagen was cross linked with keratin (a protein extracted from wool, bird feathers, skin or hair) showing an improvement in the thermal resistance and the mechanical properties of the collagen-based film [53]. In another study, collagen was used in tow formulations, namely with chitosan and with soy protein isolate. The tow composites showed good properties for applications, with slight differences. The one with chitosan showed higher elongation at break point and water vapor permeability, meanwhile the collagen/soy protein isolate showed higher tensile strength. A collagen alone film showed high resistance to heat when compared with the tow composited samples [54].

Egg white is an important, versatile and cheap source of protein. It has been used in composites [55] or for bioplastic [56]. In research, egg white film was obtained by extrusion and compared with commercial polylactic acid-based film. The comparison showed that egg white-based film is more resistant to breakage, to heat and to oxygen, and showed similar transparency, lightness and color [55].

A myofibrillar protein, surimi, isolated from muscle (chicken, beef and pork) proved to be suitable for biodegradable films obtained in acidic conditions. Reports showed good mechanical strength but, as a disadvantage, it has a bitter taste [57].

### 3.2. Vegetable Protein-Based Packages

Vegetable proteins used in food packaging are suitable for vegan diets and bring several advantages such as active ingredients, large diversity, and economic sustainability [58]. When adding plant extracts or plant byproducts into protein-based food packages, an increased bacterial protection can be registered due to the presence of phenolic compounds [7].

The main protein found in corn is zein, which has hydrophobic, antioxidant, and antibacterial properties, and forms an adhesive film [8]. By adding sugar plasticizers into zein films, the hydrophobicity can be enhanced [49].

One method for obtaining soy protein film is by boiling soy milk in a thin pot until the film is formed, while another method is based on baking soy protein isolates on pans for 1 h at a temperature of 100 °C [8]. In a study, canola and sorghum proteins were added to soy proteins to improve adhesion [59]. In another study, a coating based on Plantago major seed mucilage and enriched with *Anethum graveolens* essential oil inhibited bacterial (*E.coli*, *S. aureus, S. pyogenes, B. subtilis, B. cereus*) and fungi (*Candida albicans, Aspergillus fumigatus*) growth [60]. In a review describing the food preservation of active films and coatings, the authors reported significant inhibition on the growth of spoilage bacteria [7].

The authors obtained significant reductions in the growth of spoilage microorganisms and achieved 18 days of storage by using the active coating, whereas the shelf life of control samples was 6 days [7].

Another agro-food by-product with promising reuse potential due to the significant content of proteins, fibers and phenolic compounds is the malt spent grain biomass generated in the brewing process [61]. Due to its low cost and high availability, brewers’ spent grains proteins could be considered as a cheap alternative for the preparation of biodegradable film with antioxidant activity. In this sense, Proano et al. [62] investigated the potential of developing brewers’ spent grain protein films (casting protein dispersion) with active packaging properties. Of all the tested parameters (different pH and plasticizers levels), the films prepared at pH 2 and plasticized by polyethylene glycol (PEG) exhibited enhanced mechanical properties. In a similar study, Lee et al. [63] demonstrated that the incorporation of chitosan into the brewers’ spent grain protein resulted in a composite film with good antioxidant and antimicrobial activities.

Edible coatings can be formed by treating the grass pea flour suspension with microbial transglutaminase (mTGase) using a small quantity of glycerol (8%) as a plasticizer. The enzyme treatment has a small influence on the typical protein agglomerate size, by decreasing the particle dimension; meanwhile, the treatment with mTGase does not influence the zeta-potential and the polydispersity index of the resulting film forming solution. While the microbial transglutaminase is present, the film opacity is eight times smaller than the non-transparent polypropylen and bigger by seven times than the one produced by the transparent cellulose triacetate, the grass pea flour being slightly transparent, which is shown by the optical analysis. The SEM analyses of the coating surface and the cross-section, proven by morphology research, shows that mTGase offers a constant and soft structure. The presence of the microbial transglutaminase created bioplastics with an increased extensibility, which were less hard and more resistant, in terms of the mechanical properties [64].

According to Dilara Konuk Takma and Figen Korel, 2018, an active packaging film was made with coatings of alginate, antimicrobial chitosan and incorporated black cumin essential oil. The chicken breast was stored at 4 °C over 5 days, time in which the film demonstrated a lower color change, lower microbial growth and fewer pH changes. To give fresh and safe meat products, black cumin essential oil can be used in active packaging due to its antimicrobial activity against E. Coli, a Gram-negative bacteria which is responsible for foodborne disease; it is bacteria especially found in the raw meat foods [65].

In another study of Badr et al., 2014, the antimicrobial efficiency of edible films made from whey protein, and incorporated with 1–2.5% thyme, cinnamon and cumin essential oils on fresh beef was evaluated. After the storage of this meat at 5 °C for 12 days, it was observed that the sliced meat containg thyme essential oil had a stronger inhibition on the bacteria as a result of a higher antimicrobial activity under these parameters. The results showed that whey protein edible film which contains 2.5% w/w of cumin, thyme and cinnamon essential oil can double the shelf life of fresh beef meat when stored under refrigeration [66].

Another type of edible film was prepared from a composition of alginate-clay by adding some essential oils (cumin, marjoram, coriander, cinnamon, clove and caraway) and its antimicrobial activity was evaluated on rainbow trout slices. The maximum inhibitory effect was demonstrated by marjoram essential oil, followed by the clove and cinnamon oil. Furthermore, the results showed that the film which contained alginate-clay with 1% incorporated marjoram essential oil delayed the development of *L. monocytogenes* during 15 days under refrigerated storage with a total of 6.23 log CFU/g (colony forming units), while in control samples a 7.38 log CFU/g (*p* < 0.05) was reached. As a conclusion, the intensity of the antimicrobial activity has the following order: marjoram > clove > cinnamon > coriander > caraway > cumin essential oil [67].

It is known that some types of quinoa proteins are used to create edible films and presented remarkable consequences on their physical properties (water vapor permeability, water sorption, roughness and solubility). Being cross-linked with transglutaminase helped with the improvement of the edible films’ properties. In addition, the quinoa protein variety combined with its proteins profile is directly connected to the interactions between proteins and transglutaminase [68].

The association of the transglutaminase with the lysine of wild quinoa and quinoa Pasankalla is shown in a study presented by Escamilla-García et al., 2019. The lowest solubility (14.02 ± 2.17%, *w*/*w*) was shown by the mixture of chitosan:wild quinoa (1:5, *w*/*w*). The water vapor permeability was different and varied because of the composition of the mixture. The water vapor permeability of the chitosan:quinoa protein varied from 2.85 to 9.95 × 10^−11^ g cm Pa^−1^ cm^−2^ s^−1^, in the absence of transglutaminase. When transglutaminase was added to the mixture the range was reduced to 2.42–4.69 × 10^−11^ g cm Pa^−1^ cm^−2^ s^−1^. The film surface roughness was reduced from 8.0 ± 0.5 nm to 4.4 ± 0.3 nm by adding transglutamminase to the chitosan:quinoa Pasankalla composition. Regarding the sorption isotherm, the added transglutaminase enhanced the stability of the chitosan:quinoa wild films (monolayer (Xm) = 0.13 ± 0.02%). Consequently, the enumerated physical properties showed a much higher improvement when the edible films had a higher quantity of cross-linking. The interactions between the proteins that were caused by the amount of transglutaminase depended on profile and the protein source [69].

In the research presented by Porta et al., 2015, it was proven that the surface of films is smoother and more compact in the presence of transglutaminase than when prepared without this enzyme. Studies about surface roughness show significant difference between films’ preparation with (Rq ¼ 41.1 ± 1.2 nm) or without (Rq ¼ 84.4 ± 1.5 nm) transglutaminase. In addition, the control films prepared with transglutaminase enzyme are much more homogeneous, resistant, firm, and permeable (oxygen 700-fold and carbon dioxide 50-fold), than the control films prepared without the enzyme which have irregular zones in the films’ cross-sections [70].

Another study of the same topic included bitter vetch protein films but with spermidine, without or with a low quantity of glycerol and it was demonstrated that, by increasing the plasticizer amount, the tensile strength was gradually reduced. It was shown that the film extensibility and flexibility were improved by the spermidine, by helping the reduction in glycerol-dependence of the intermolecular forces beside the chain proteins, and also by acting as a plasticizer by ionic interaction with proteins. In this way, spermidine can be considered a second plasticizer because of its capacity to improve glycerol plasticizing action. The films which contain spermidine were analyzed under a microscope and it was confirmed that the matrices are more uniform, cohesive and compact [71].

**Figure 2:**
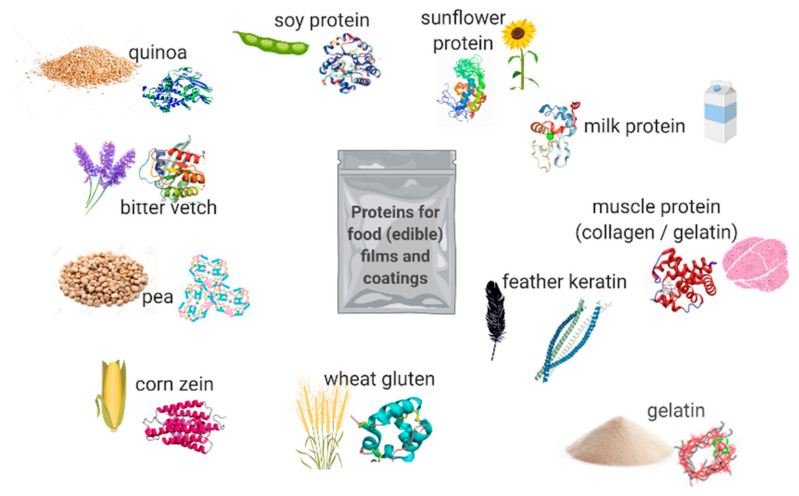
Most utilized materials for protein-based (edible) films and coatings.

Figure 2 shows the most utilized proteins for food films and/or coatings.

## 4. Protein-Based Films and Coating Functionalization

In the agro-food industry, proteins, polysaccharides, and lipids represent the highest amount of macroelements present in crops and waste streams. In plant and animal tissues, proteins can be found in a large variety of structures and accomplish various functions. Among these functions, probably the most known are related to their involvement in biochemical reactions and the building of tissues. The excellent and wide range of functional properties of proteins will fill the need for high-performance renewable materials. Chemical, enzymatic or physical modifications and treatment of protein films as well as the preparation together with other hydrophobic polymers can have a positive influence on the mechanical strength of protein films and on their poor water vapor resistance. For the preparation of functionalized (edible) films and coatings, the most utilized proteins are casein, gelatin, wheat gluten, soy protein or zein [72]. In the processing technique, coatings are applied to food products by liquid methods and films are obtained as solid laminates and then applied to food products [73]. Regarding the biodegradability, protein-based food packages are among the most feasible ones [74]. Low price and sustainability are the most important aspects from the industrial point of view [75]. The inclusion of different components like natural antioxidants improves the antioxidant properties or strengthens the protein networks.

The insertion of functional groups in the amino acid side chain of proteins allows covalent and non-covalent crosslinking. Chitosan derivatives acts as non-covalent crosslinking agents based on hydrogen bonding with plant proteins like whey [46]. The crosslinking leads to an increase in the film’s insolubility and elongation and an increase in the surface hydrophobicity with a contact angle larger than 90°. These effects on the film properties are possibly caused due to conformational change of the proteins after the crosslinking.

An important functionalization of the protein-based films is described by Cano et al. They analyzed the antioxidant activity of tannins incorporated in protein-based packages. Furthermore, tannins from different sources (white peel grape, red peel grape, from oak bark, guava leaves etc.) have also proved to have antimicrobial activity. The addition of tannin make the package less soluble [76].

An emerging technology—cold plasma—is used for the protein film and coating properties modeling, namely by improving the adhesion properties [74]. The influence of different plasma treatments as a method for modification is investigated and described by Romani et al. [77].

Thermal treatment of food proteins, e.g., from yellow peas, causes physical and chemical changes to their structures due to the fact that proteins rearrange which cause their secondary and tertiary structures to unfold due to the breaking of hydrogen bonds. This effect leads to an increase in surface hydrophobicity [78].

Incorporation of montmorillonite clay together with citric acid increased the barrier properties and leads to a sequential decrease in the physicochemical quality loss in processed apples [79]. The addition of cellulose nanocrystals (CNCs) can lower the moisture amount of the protein-based package by disintegrating the hydrogen bonds between the proteins’ amino groups and the molecules of water. Additionally, the filling effect of the incorporated CNCs make the film more rigid [80].

The incorporation of natural antioxidants into protein films has an effect on the antioxidant activity. The incorporation of mango kernel extracts (MKE) in soy protein isolate (SPI) and fish gelatin (FG) films maximizes the antioxidant activity. The FG films showed improved thickness, higher tensile strength, and ensured more transparency, meanwhile, SPI film showed higher antioxidant activity and improved water barrier properties [18]. Into a whey protein-based active film, rosemary and thyme extracts were incorporated and the antioxidant activity of the extracts was evaluated [81]. Table 2 sums up some functionalization and proposed applications of the protein-based packages.

## 5. Antioxidant, Antimicrobial/Antifungal Activity of Protein-Based Films

**Figure 3:**
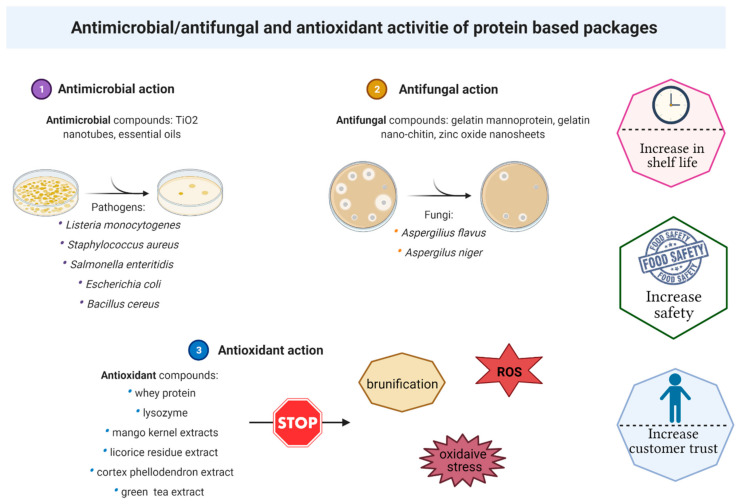
Protein-based packages and their additions of antimicrobial/antifungal and antioxidant activities.

Oxidation and microbiological contamination are the main processes blamed for food spoilage and food-borne illnesses. There are plenty of studies (Table 3) on protein-based films and coatings that sustain bioactive compounds (with antioxidant and antimicrobial activity) incorporation into the package in order to obtain an extended shelf life of the food product (Figure 3).

The conveniences of this practice are given by the fact that the biomolecules incorporated into the film or coating, (i) do not influence the product teste, (ii) are released in a controlled manner and (iii) may ensure less additives are inserted (antioxidants, preservatives) into the product. Moreover, biodegradable and inexpensive make characteristics them even more suitable.

Bioactive compounds may have various sources and thus various modes of action.

## 6. Physicochemical Properties of Protein-Based Packages

The physical and chemical properties of protein-based (edible) films and coatings should be correlated with their plasticization properties but also with characteristics such as the mechanical properties, thickness, moisture content, water vapor permeability, sensorial properties and suitability for the environment. In a study presented by Bourtoom T., 2008, the parameters which affect the characteristics of the films prepared from mungbean protein were described. In this research, it was found that the effect of the heating temperature and pH were the most important on the properties of the edible films correlated with the heating time. At pH 9.5 at 75 °C (5.70–6.51 MPa) the highest point for the tensile strength was reached and the lowest was registered for the elongation at break (32.06–40.08%). In these conditions, the protein (19.26–27.00%) and films’ solubility (37.53–39.43%) and water vapor permeability (11.37–16.91 g·mm/m^2^ day kPa) were at their lowest. In addition, by increasing the heat temperature and pH of the film solution, the color became more yellow and darker [98].

### 6.1. Mechanical Properties

In order to maintain the integrity of the food product during the shelf life, the mechanical strength [99] and extensibility [100,101] of the protein-based film or coating are of high importance. Processing, packaging, and storage are the first tests that can give indications about the package resistance and ability to protect foods [102,103]. The presence of structural deficiencies, the structural adaptation of the major components of protein-based packages, the distribution and density of the intra- and intermolecular interactions between the polymer chains, and the type and amount of plasticizers have been reported to affect the mechanical profile of the films or coatings [73,104].

The plasticizer glycerol is an effective parameter that determines the mechanical properties of protein-based films for food protection. It reduces the intermolecular forces between polymers by reducing the tensile strength and increasing the elongation at break [105]. Moreover, the presence of cavities and holes could decrease the film or coating flexibility and tensile strength. Therefore, protein-based packages must have good mechanical properties to protect the food, resist food processing, handling, and storage stress. Gialamas et al. [106] demonstrated that no significant changes were observed in tensile strength, elongation at break, and modulus of elasticity of sodium caseinate edible films containing probiotics, because of the relatively low mass of the added probiotics with little impact on the mechanical properties of protein-based edible films. Consequently, cellulose-based edible films, with greater mechanical resistance, are a little more sensitive to the incorporation of the probiotic cells.

Formulation of edible films with glycerol-plasticized thermoplastic pea starch (TPS)/ microcrystalline cellulose (MC) and TPS/carboxymethyl cellulose (CMC) mixtures, was conducted using a screw extruder followed by analysis of the biodegradable polysaccharide (thermoplastic starch/cellulose derivatives). Following the studies, the glass transition temperature and the coefficient of elasticity were improved. Thermostablity was increased by the microcrystalline cellulose composition, while the carboxymethyl cellulose decreased this parameter. Elongation at break at low water content (13%) and the tensile stress were expanded by the MC and CMC mixtures. A good connection between starch and cellulose derivatives is responsible for the changed parameters described previously [107].

### 6.2. Thickness

A correlation with the thickness of the protein-based film and with the other characteristics such as water vapor permeability, transparency, and mechanical properties can be made. The preparation method and working parameters (e.g., pH) and the drying conditions (e.g., temperature) are dirrect influence the thickness of protein-based films [108,109].

Soukoulis et al. [110] stated that no significant impact on thickness was observed by the addition of *L. rhamnosus* GG cells into probiotic-containing edible film. Conversely, Soukoulis et al. [111] in another study, demonstrated that the inclusion of probiotics in film-forming solutions changed the film thickness. A direct influence of the addition of probiotic cells into the protein-based film and its thickness is sustained also in another study where *L. casei* was incorporated into whey-based film [112]. Studies are not necessarily consistent in this direction. Pereira et al. report no modification in the thickness of the film when *B. animalis* or *L. casei* was added to whey edible films [113].

Thus, this property seems not to affect the optical characterstics, or the water solubility [114]. No correlation between elongation at break and the relaxation coefficient of fish myofibrillar protein-based package and film thickness was observed; meanwhile, break resistance was dirrectly influenced by this property.

### 6.3. Water Vapor Permeability (WVP)

The WVP is directly influenced by many factors (e.g., polymer chains mobility, thickness, film integrity), but the most mentioned in the literature are the kind and the amount of plasticizer. The major convenience of increased water vapor permeability is the improved solubility of the protein-based films. This characteristic can be directly correlated with a better release of bioactive compounds, from functionalized or active packages, which could be incorporated into the protein-based film [14,18].

### 6.4. Moisture Content

Together with the water permeability, the moisture content may be dirrectly correlated with the package capacity to prolong the product shelf life [115] and the function of active ingredients incoroprated into the film or coating. For example, the moisture content after drying influences the rate of probiotic viability during long storage periods and simplifies the melting of edible films in the mouth [38].

### 6.5. Sensory Properties

The sensorial evaluation of biopolymers-based films is essential for success on the market. It is well known that the incorporation of bioactive compounds into food products can alter the sensorial acceptance significantly. In such a manner, by incorporating bioactive compounds into biopolymers-based films, the sensory properties of biopolymers-based packages will be unaffected [14,39,40,116].

For example, the color directly influences the consumers’choice for food products. Thus, an important property of the packages is transparency. Therefore, the techniques for obtaining protein-based packages should take into consideration the influence of the light that is passing through the protein-based films [5,117].

For example, in probiotic functionalized protein-based packages, an upper layer is formed by the active cells. This behavior can be explained by the difference between the density of probiotic cells and the polymer solution during the drying stage and is negatively affecting the transparency [118]. Ly et al. [119] state that this disadvantage can be overcome by reducing the viscosity of the film-forming dispersion. Another important aspect is the surface charge of the probiotics. In the protein coating formation process, these cells interact (electrostatic) with charged polymers. Nevertheless, protein films’ and coatings’ optical properties are influenced by many factors. Among these, we can count crystallites’ mean size and crystallinity, the amount of plasticizer and its type, structural conformation, the refractive index, and compatibility of the film [120].

## 7. Safety Issues

There are many ways in which food quality can be altered causing spoilage of the products, the most common reasons being oxidation and microbial growth. To make food packages more reliable, scientists began using the packaging system not only to provide critical information about the product, but also to be a line of defense against microorganisms and oxidation. Active packaging begins to be used on a larger scale simply because it helps improve the products’ shelf-life and quality.

A common procedure to overcome the simple barrier of (protein-based) films and coatings, and bring more value to the safety aspect of the package, is to incorporate active materials. These procedures develop a new class of packages—active packages [117]. The safety issues are controlled by the active protein-based films and coatings using mechanisms such as decreasing the microbial development, delaying oxidation through antioxidant compounds and decreasing moisture migration. By implementing these active components, the food quality, shelf-life, and freshness are also improved [20].

Intelligent packaging can overcome safety concerns, as monitoring systems can give information about the status and quality of a product directly from the package, and therefore helps to reduce food waste and spoilage [121]. The most common indicator used in this kind of packages is the change in color, and among the quality change the microbial activity is the most monitored one [122].

Various researchers discuss whey protein-based films’ property of having a relatively low oxygen permeability [123]. This fact make them potentially useful for coatings or other film materials used for oxygen-sensitive food products [124].

Minimizing the growth rate of foodborne pathogens by using antimicrobial agents in packaging material could extend the shelf life of packaged foods. In the last decade, food additives, preservatives, were the main option for food shelf-life extension. Consumers and healthcare institutions’ concerns about additives levels led to the development of innovative antimicrobial films and coatings application methods.

An antimicrobial protein or non-protein packaging system is developed by the incorporation of antimicrobial fillers directly into the films, by coating the packaging films with antimicrobial compounds, or by using polymers that have natural antimicrobial features. Afterwards, the antibacterial agent is slowly released on top of the food surfaces. The slow release system helps retain a sufficient concentration of the antibacterial agent, ensuring antimicrobial protection during the product shelf life [125]. Frequently utilized antimicrobial agents are herbs and nanoparticles [126,127]. The typical herbs such as thyme, oregano and tarragon contain caffeic acid [128], which has a strong effect against pathogenic bacteria, viruses, and fungi. Flavones have phenolic structures with one carbonyl group. These kinds of compounds are synthetized by the plants in order to protect them against microbial attack. Due to this fact, their action is efficient against a broad range of pathogens [125,129].

Nanoparticles and nanostructures of silver or gold inhibit the growth of foodborne pathogens due to their broad-spectrum antimicrobial activities. Moreover, nano-silver and nano-gold could catalyze the absorption and decomposition of ethylene emitted from fruit metabolism, which is blocking the ethylene and thus ensure prolonged shelf life [130]. An increase activity of the nano-functionalized package can be explained by the fact that, for example, ZnO nanoparticles directly interact with the food, significantly increasing the shelf life [131,132]. Nanotechnology has is gaining field not only in medicine but also in cosmetics and food sectors [133]. Due to their nanosized structure, their exact behavior in the human body (i.e., edible packages containing nanoparticles) is under safety concerns. Recent analyses regarding the toxicity, behavior, and long-term use of nanoparticles in food systems underline the urgent need for more studies. Nanoparticles, in any food system, are only allowed to be used, in Europe (EU), if they are stated to be safe in Annex I of the 10/2011 EU regulation [134]. Safety concerns rise especially in the cases of edible packages, intelligent and active packages where the nanoparticles are released and come into contact with the food on purpose. Authorities and consumers’ concerns are related to nanoparticles’ toxicity and accumulation in the body. Burger et al., in their cytotoxicity study, found that all investigated nanostructured lipid carriers are biocompatible with skin cells, but some of them are sensitive to UV irradiation [135].

Furthermore, in any new formulation, the components (new protein sources, extracts) must be verified as being accepted for contact with food products or for ingestion, in the case of edible packages.

Anyway, an important issue regarding food safety is protein-based edible films and coatings made from edible ingredients. In this sense, it is imperative that edible films and coatings ingested together with the food product be safe for consumption, with no health risk involved [8].

## 8. Economic Perspectives

Due to the fact that the global population is in continuous growth and also because of the increase in food consumption, in the next 20 to 40 years, there will be a higher demand for food [136,137]. Economical and financial aspects such as high prices of land and resources such as water, and energy will negatively impact farmers and the food industry [138]. Another important aspect in this circle of producers, industry and consumers is the environment. It is highly important to minimize the impact of the food system on the environment [139]. Humanity’s wellbeing and global resources are threatened by the effects of climate change caused by pollution [140,141]. Despite the fact that a better organization and a more efficient and equitable utilization of the food will solve some of the problems, urgent and innovative solutions need to be applied in order to decrease the amount of food and food-related waste [142]. A solution for the reduction in food packaging waste was the creation of family-sized packages. A higher amount of food contained a single package, and a good discount price are probably the only advantages of these kinds of deals, but then society is facing an overestimated consumption and the purchase of an unnecessary amount of food [143,144].

It is true that this kind of package bring financial benefits to the producers (higher production and decreased cost of manufacturing); thus, this trend is generally applied [145]. If we are to analyze the presented situation from several perspectives, we must talk about the food wastage/spoilage linked to this behavior and also about the implications in the health systems [146]. According to Food and Agriculture Organization of the United Nations (FAO) no less than 1.3 billion tons of food is wasted every year globally [20]. Indeed, there are strategies approached by World Health Organization (WHO) and by governmental organizations in order to reduce food waste and in order to increase the consumer awareness about the fact that it is not quantity that is really important, but nutritional and functional quality [147]. In this direction, more programs need to be implemented regarding the packages’ functionalities and implications in keeping and boosting food quality. Last but not least, food package reutilization and reconversion need to be highlighted in the consumer’s view [148].

Carrying out strong food waste prevention strategies such as changing the acceptance criteria for fresh produce instead of weak prevention strategies could fulfil more in terms of long-term sustainability [149,150]. Even the strategies (e.g., food banks) already implemented for reducing food and food packaging waste need to be improved. From a food security point of view, the foods closer to the end of their shelf lives could indicate an increased risk for food-borne disease or may have decreased quality. Usually, these kinds of food are sold with high discounts or given away to socioeconomically disadvantaged groups [151], and no sufficient information about food urgency to be consumed nor about package waste management is delivered.

Certainly, among consumers, there is a popular belief that food packaging represents an additional economic and environmental cost, an unnecessary solid waste rather than a valuable means for waste reduction. According to Patil et al. (2004) [151], 75–90% of consumers agree that discarded packaging is a greater environmental issue compared to wasted food [143].

Many economic and social benefits will be brought about by reducing the environmental cost of packaging waste. Substitution of petroleum-based polymers with bio-based polymers leads to reduction in greenhouse gas emissions and non-renewable energy use [152].

Among all other properties, the cost is a significant factor in the consumer’s assessment of edible films. Nowadays, edible films cost 10–50 times more than conventional packaging (plastic), which is derived from natural gas or petroleum [5]. In the few last years, in Europe the price for conventional packages increased by 30–80%, mainly due to growing petroleum cost, which leads to the development of eco-friendly packaging materials; in particular, legislation from Japan and the UK enhanced the improvement of these types of films [153]. On the other hand, for the development the edible films, the cost is not an impediment, because these are applied in the food industry with a particular aim, namely, to add value to the final product [154]. In the long-term, edible films based on proteins can reduce food waste, which is a real challenge currently (e.g., the food waste in the United States of America is around USD 161 billion/year and 95–115 kg/year per capita higher than in Europe); food safety will be higher and the confidence of the customer greater.

Even if synthetic packages have many advantages (great feasibility for industrial production, apparent and short-term economical advantage), their chemical nature is blamed for environmental problems [155], and alternatives, even more profitable ones will emerge soon.

Integrated strategies, the development of new and innovative technologies, and more innovation in materials sciences may be the light out of this tunnel. For example, it is estimated that nanotechnology, and its applications in food packaging, will impact the global economy [156]. Diminishing the conventional food packaging, without compromising food security, is a big challenge. From an economic point of view, shelf life extension would allow minimization of transport costs, generating economies of scale and downsizing the minimal scale of production, which is especially beneficial for small companies [157]. Thus, this desiderate needs to activate the development and production of functionalized food packages.

Another interesting aspect is the reshaping of actual food packages. Most of them are oversized. Scientists and food packages manufacturers can find alternatives (e.g., reducing the thickness) that may significantly impact food packaging waste and all the economic aspects derived from these strategies, without affecting the security of the foods, nor the marketing aspects [157].

Magrassi states the fact that the recycling rate has been raised from 70 to 95% and datasets have been implemented including environmental benefit of recycling and costs the allocated to the production of the recycled steel and glass [158].

## 9. Conclusions and Future Perspectives

The development of plastic replacement packages is beginning to be a reality in the food industry. In order to meet the environmental need—no wastes—natural, feasible, and available materials are in the spotlight of researchers and producers. Proteins from various sources (vegetables, animals, by-products) have been proven, as can be concluded from studies described in this review, as one of them.

While reading this review, it is possible to realize that a limitless area is in front of us. Protein-based packages, either films or coatings, may be obtained by using different techniques and different combinations. The development of plastic replacement packages is beginning to become a reality in the food industry.

The current trend is to functionalize (for antioxidant, antibacterial, antifungal properties) these packages and make them intelligent, and/or active. In order to achieve these properties two pathways are used: (i) design of controlled release packaging that incorporates natural or synthetic active molecules (polyphenols, nanoparticles, etc.) or (ii) designing a package that changes along with the product status (pH, microorganisms, etc.) and informs the customer.

Protein-based packages are a great alternative to conventional food packages. At this moment, they are more expensive alternative but are much more valuable and cost-efficient in terms of long-term properties such as biodegradability and eco-friendliness, vegetarianism, suitability, and consumer acceptance.

## Figures and Tables

**Table 1:** Protein-based packages formulations and attainment methods.

Formulation	Attainment Method	Characteristics	References
Sodium caseinate, bee wax and fatty acids	Film-forming emulsions	↑ rigidity ↓ whater permeability ↑ laminate-like structure	[34]
Gelatin, soluble starch and polyols	Drying casting aqueous solutions	↓ elasticity and tensile strength in samples high in water, glycerol or sorbitol	
Bovine hide and pigskin gelatin	Casting technique	↓ puncture force by sorbitol addition ↑ water permeability with the gelatin content	[35]
Gelatin vs gelatin and casein	Cross linked with transglutaminase	↑ elongation no modification in tensile strength and water vapor barrier properties	[36]
Whey protein concentrate and sodium caseinate	Casting technique	↑ mecanical and tensile strength ↑ resistance to puncture no modification in elongation at break, water vapor barrier properties and moisture content	[37]
Milk proteins	Transglutaminase-catalyzed polymerization	↓ moisture transfer ↓ whater vaopr resistance	[38]
Chitosan–whey protein	Casting technique	good moisture content ↓ water activity	[39]
Sunflower protein	Casting technique	↓ water solubility ↓ glass transmising temerature	[40]

**Table 2:** Overview of the functionalization and proposed application of protein-based packages.

Functionalization/Material	Outcome	Application	References
Whey protein and gluten protein films treated with cold air and argon plasma	↑ tensile strength ↑ roughness of whey protein-films ↓ roughness of gluten protein-films ↓ gas permeability (oxygen) ↑ stability of whey protein-films against water ↓ stability of gluten-films against water ↑ hydrophilicity of whey protein-films	Biodegradable food packaging	[74]
Whey protein films (WPI) activated with low quantities of water soluble chitosan (WSCh)	↓ film‘s solubility ↓ film‘s elongation mechanical resistance barrier to water vapor ↑ surface hydrophobicity ↑ antifungal properties	Food packaging	[46]
Soy protein isolate (SPI) material with integrated polyethyleneimine (PEI) an metal ions Cu(II) or Zn(II)	↑ tensile strength mechanical properties can be tuned ↑ material stretchability, ↑ self-healing capability ↓ restoration time ↑ antibacterial activity	Tissue regeneration, gene delivery, packaging, adhesives, food packaging	[75]
Preparation of shrimp shell waste protein-based films modified with oolong tea, corn silk and black soybean seed coat extracts	↑ thermal stability ↑ barrier against UV light ↑ antioxidant activity	Biodegradable films for active packaging	[82]
Incorporating cellulose nanocrystals (CNCs) and pine needle extract (PNE) into soy protein-based films.	↓ moisture content ↓ elongation at break ↑ tensile strength ↓ water vapor permeability ↑ antioxidant activity	Active food packaging material	[80]
Incorporating montmorillonite and citric acid into whey protein isolate films to preserve fresh-cut apples	↓ enzymatic browning ↓ loss of apple quality ↑ shelf-life	Active food packaging	[79]
Fish protein-films treated with cold plasma and carnauba wax coating	↑ tensile strength ↑ barrier properties ↓ water vapor permeability ↑ adhesion properties	Food packaging	[77]
Fisch protein-films treated with glow discharge plasma	↑ elongation at break (*) ↑ tensile strength (*) ↑ color properties (*) ↑ barrier properties (*) (*) dependent on plasma treatment time	Food packaging	[83]
Incorporating mango kernel extract into soy protein isolate films and into fish gelatin films	↑ thickness ↑ tensile strength ↑ transparency ↑ antioxidant activity ↓ water vapor permeability (*) (*) in soy protein isolate films	Food packaging	[18]
Incorporating rosemary and thyme extracts into whey protein films	↑ antimicrobial activity	Active food packaging	[81]
Adding tannins to caseinate films or gelatin films	↑ antioxidant activity (*) ↑ antimicrobial activity (*) ↓ water solubility (*) ↓ water vapor permeability (*) ↓ stretchability (*) ↑ thickness (*) (*) only for caseinate films	Active food packaging	[76]
Incorporating yerba mate and white tea extracts into furcellaran/whey protein films	↓ water solubility (*) ↓ water vapor permeability (*) ↓ water content (*) ↑ shelf-life ↑ antimicrobial activity ↑ thermal stability ↑ puncture strength (*) ↑ modulus elasticity (*) ↓ elongation at break (*) (*) only for yerba mate extract	Edible active food packaging	[84]

↑ increase, ↓ decrease, – no increase.

**Table 3:** Antioxidant, antimicrobial and antifungal activity of protein-based films and coatings.

Film/Coating	Formulation	Antioxidant Capacity	Antimicrobial/Antifungal Activity Against	Reference
Edible coating	whey protein isolate whey protein concentrate hydroxypropyl methylcellulose beeswax or carnauba wax	decrease enzimatic browning (just for the whey protein-based coating)		[85]
Film	Polyvinyalcohol with lysozyme	-	*Micrococcus lysodeikticus*	[86]
Film	mung bean protein pomegranate peel (0, 2.5, 12.5, and 25% w/w)	13.88 mg GAE/g (gallic acid equivalents) (25% pomegranate peel)	*Escherichia coli O157:H7* *Listeria monocytogenes*	[87]
Film	soy protein isolate with cortex *phellodendron* extract (0, 10, 12.5, 15, 17.5, 20, 22.5% w/w)	14.87 mg GAE/g (22.5% *phellodendron* extract)	*Staphylococcus aureus* ↓ *Escherichia coli*	[88]
Film	soy protein isolate fish gelatin mango kernel extracts	3.77 μg GAE/g film	-	[18]
Film	soy protein isolate licorice residue extract (10, 30, 50, 70 g/kg)	20% higher than in the control	-	[89]
Film	distiller dried grains with soluble (protein) green, black and oolong tea extract (0.1, 0.3, 0.5%)	all 0.3% samples had over 50% higher antioxidant activity than control	-	[90]
Film	soy protein isolate chestnut (*Castanea mollissima*) bur extracts (20, 50, 80, and 100 g/kg)	at least 20% higher than the control	-	[91]
Film	fish myofibrillar protein catechin–Kradon extract	at least 40% higher than the control	-	[92]
Coating	Whey protein TiO_2_ nanotubes	over 50% higher than the control	*Listeria monocytogenes* *Staphylococcus aureus* *Salmonella enteritidis* *Escherichia coli*	[93]
Film	cassava starch and whey protein rambutan peel extract cinnamon oil	over 30% higher than the control	*Bacillus cereus* *Staphylococcus aureus* *Escherichia coli*	[94]
			*Aspergilus niger* CGMCC	[95]
Film	soy protein isolate plant-sourced cinnamaldehyde zinc oxide nanosheets	-		
Film	gelatin nano-chitin	-	*Aspergillus niger*	[96]
Film	gelatin mannoprotein (extracted from *Saccharomyces cerevisiae* cell wall)	-	*Aspergillus flavus* binding aflatoxin B1	[97]
